# From the Front or Back Door? Quantitative analysis of direct and indirect extractions of α-mangostin from mangosteen (*Garcinia mangostana*)

**DOI:** 10.1371/journal.pone.0205753

**Published:** 2018-10-15

**Authors:** Edison Eukun Sage, Nashriq Jailani, Azney Zuhaily Md. Taib, Normah Mohd Noor, Md. Ikram Mohd Said, Muntaz Abu Bakar, Mukram Mohamed Mackeen

**Affiliations:** 1 Chemistry Programme, Centre for Advanced Materials and Renewable Resources, Faculty of Science and Technology, Universiti Kebangsaan Malaysia, Selangor, Malaysia; 2 Institute of Systems Biology, Universiti Kebangsaan Malaysia, Selangor, Malaysia, UKM Bangi; 3 Malaysia Genome Institute, Jalan Bangi, Selangor, Malaysia; College of Agricultural Sciences, UNITED STATES

## Abstract

The pulp and pericarp of mangosteen (*Garcinia mangostana*) fruit are popular food, beverage and health products whereby 60% of the fruit consist of the pericarp. The major metabolite in the previously neglected or less economically significant part of the fruit, the pericarp, is the prenylated xanthone α-mangostin. This highly bioactive secondary metabolite is typically isolated using solvent extraction methods that involve large volumes of halogenated solvents either via direct or indirect extraction. In this study, we compared the quantities of α-mangostin extracted using three different extraction methods based on the environmentally friendly solvents methanol and ethyl acetate. The three solvent extractions methods used were direct extractions from methanol (DM) and ethyl acetate (DEA) as well as indirect extraction of ethyl acetate obtained via solvent partitioning from an initial methanol extract (IEA). Our results showed that direct extraction afforded similar and higher quantities of α-mangostin than indirect extraction (DM: 318 mg; DEA: 305 mg; IEA: 209 mg per 5 g total dried pericarp). Therefore, we suggest that the commonly used method of indirect solvent extraction using halogenated solvents for the isolation of α-mangostin is replaced by single solvent direct extraction using the environmentally friendly solvents methanol or ethyl acetate.

## Introduction

*Garcinia mangostana* L. (Guttiferae/Clusiaceae) commonly known as mangosteen is a tropical economic plant native to Southeast Asia that is believed to have originated from Peninsular Malaysia. It is planted in Southeast Asia and other tropical regions namely India, Sri Lanka, Northern Australia and tropical America [1] and can be differentiated (according to their respective localities) by careful examination of the leaves via Fourier transform-infrared coupled with chemometric analysis [2]. Its economic value arises from its perception as a superfruit [3] that is dubbed the “queen of fruits” in Southeast Asia [4]. Its fruit predominantly comprises an inedible dark purple or red pericarp (> 60%) that encases an edible succulent pulp [5]. The pericarp has been used as a natural dye and has recently shown potential as a dye-based sensitiser in solar cells [6]. Mangosteen seeds are recalcitrant due to its low temperature intolerance and sensitivity toward desiccation [7]. The latter part of the fruit is popularly consumed directly/freshly or as processed food products such as syrup, canned drinks and cubes, marmalade as well as health-related dietary supplements [8]. More recently, the pericarp that in the past was typically discarded as waste has gained ever increasing attention for its purported health-related properties thereby creating a secondary economic demand for it. This trend has been bolstered by the long-standing use of the pericarp in traditional medicine and the growing scientific studies on its potent biological activities. In traditional medicine, the extract of mangosteen pericarp has been used to treat a variety of ailments such as fever, diarrhoea, dysentery, menstrual cramps, urinary tract infections and many other conditions [9, 10]. Recent studies on the pericarp extracts of mangosteen have shown inhibition and modulation towards, for example, advanced glycation, α-glucosidase activity, immune response and hyperglycaemia performed in cell-free and cell-based studies including whole animals and humans [11–13]. Therefore, the increasing economic value of this plant has resulted in its commercial cultivation especially in Southeast Asia for the regional and international markets [14, 15].The pericarp of *G*. *mangostana* is saliently characterised by the abundant presence of prenylated xanthones of which α-mangostin is the major constituent. α-Mangostin has been reported to be stable under normal and stress conditions [16, 17]. This metabolite is highly bioactive that has been attributed with multiple *in vitro* biological activities ranging from the inhibition of pathogen- to metabolic-related activities such as antimicrobial [18], cytotoxic [19], antitumour [20], anti-inflammatory [21], antimalarial [22], antiviral [23], antimycobacterial [24], antioxidant [25], antiglucosidase [12] and antileptospiral [26] activities. The *in vivo* antihyperglycaemic, anti-inflammatory, antioxidant and antitumour activities of α-mangostin applied as pure compound or as part of a food/health product prepared from the pericarp has been reported (including studies in humans) [12, 27–30].

The preparative-scale abundance of α-mangostin in the pericarp has facilitated the feasibility of these *in vivo* studies. Its high abundance and wide range of *in vitro* and *in vivo* biological activities show some parallels with curcumin, the diarylheptanoid that is highly abundant in turmeric [31,32]. The reported *in vitro* and *in vivo* bioactivities of α-mangostin necessitate the preparation of preparative quantities for further functional studies as well as commercial health-related applications. The known low bioavailability of α-mangostin (30) has been improved via incorporation into carriers such as cotton seed oil [33, 34] and poly(*N*-isopropylacrilamide)-co-2VP [35]. It has been shown *in vivo* that α-mangostin is conjugated during Phase II metabolism into mono- and diglucuronide forms [34] which would increase water solubility for excretion. The intraperitoneal injection of α-mangostin was found to be a better pharmacokinetic alternative to oral administration with a higher C_max_ (maximum serum concentration after treatment) value of 7.5 μM in mice [36].

Preparation of derivatives of α-mangostin via chemical and enzymatic syntheses or biotransformations, will expand the repertoire of structures available for biological and functional studies. The chemical derivatisation of α-mangostin followed by the assessment of biological activities has been reported in several studies [37–39]. Chemoenzymatic syntheses of α-mangostin glycosides have performed using a glycosyl transferase from *Bacillus lichemiformis* [40] and amyloglucosidase from *Aspergillus niger* [41]. Biotransformations of α-mangostin have afforded several non-glycosylated and glycosylated derivatives [42, 43]. Several unique sulfur-based derivatives of α-mangostin have been obtained via biotransformations using the fungi (*Colletotrichum gloeosporioides* (EYL131) and *Neosartorya spathulata* (EYR042)) that have yet to be synthesised chemically. Many of these chemically prepared or biotranformed derivatives have shown stronger biological activities than α-mangostin such as towards *Mycobacterium tuberculosis* H37Ra [44]. Therefore, the importance of α-mangostin and its prepared derivatives justifies improving the isolation of α-mangostin from the pericarp of mangosteen.

Isolation of α-mangostin from the pericarp predominantly involves solvent extraction methods with long processing times and the use of large volumes of organic solvents despite the recent application of some alternative methods such as dynamic ultrasonic-assisted extraction [45], supercritical fluid extraction [46] and aqueous micellar biphasic system extraction [47]. The most commonly reported preparation of *G*. *mangostana* pericarp extract employs conventional solvent extraction by soaking or distillation (viz. Soxhlet-based). Solvent extraction of *Garcinia mangostana* for the isolation of α-mangostin is performed either directly (without solvent partitioning) or indirectly using solvent partitioning. The former approach involves direct contact of the plant material in a single solvent, e.g. chloroform [12] or methanol [48] or sequentially in multiple solvents [49] whereas the latter typically involves indirect extraction via successive solvent partitioning. Indirect extraction is initially carried out using a polar alcohol solvent usually methanol or ethanol in direct contact with the plant material following by sequential liquid-liquid extraction with less polar solvents. A common workflow for indirect extraction would be the sequential solvent partitioning of the initial methanol or ethanol extract beginning with the least polar solvent, for example ethanol → hexane → chloroform → n-butanol [50] or methanol → dichloromethane → ethyl acetate → n-butanol [25]. For simpler solvent partitioning, only a single solvent, e.g. ethyl acetate [51] or chloroform [52], is employed for liquid-liquid extraction with the initial alcohol fraction.

Although direct and indirect solvent extractions have been applied to the isolation of α-mangostin from the pericarp of *G*. *mangostana*, a comparison of α-mangostin yields between both approaches has yet to be reported. Additionally, in studies reporting both extraction approaches, many continue to use halogenated solvents such as chloroform or dichloromethane which restrict their use in food, herbal and pharmaceutical applications due to toxicity [53]. Therefore, there is a need to pursue the use of green solvents that are more environmentally friendly and less toxic such as ethanol, methanol or ethyl acetate [54, 55] that are commonly used for the isolation of α-mangostin.

Although the DM extract showed the lowest concentration of α-mangostin (53.8% w/w) compared to the DEA (70.7% w/w) and IEA (66.9% w/w) extracts, it afforded the highest amount of extract. This corresponded to both the DM and DEA extracts showing the higher quantities of α-mangostin per total amount of dried pericarp used in this study (DM: 305.4 mg; DEA: 280.1 mg; IEA: 244.2 mg). Therefore, single-solvent direct extraction using the methanol or ethyl acetate is a better option than indirect extraction based on solvent partitioning for the extraction of α-mangostin. Furthermore, methanol or ethyl acetate are green solvents that can replace the halogenated solvents commonly used in many studies reporting the extraction and isolation of α-mangostin.

## Materials and methods

### Chemicals and reagents

The solvents used were of HPLC or analytical grades, and were purchased from Merck. Silica gel 60 PF254 containing gypsum 107749 was used for radial chromatography with a thickness of 4 mm. α-Mangostin was isolated in a single step from the directly obtained ethyl acetate extract (500 mg) using radial chromatography with stepwise n-hexane/ethyl acetate elution (9:1 to 5:5). The purity of the targeted α-mangostin containing fractions was evaluated by analytical TLC (Rf = 0.8 hexane:ethyl acetate = 2:8) and high-performance liquid chromatography (HPLC) (described below). Identification of α-mangostin was carried out using direct-infusion electrospray ionisation mass spectrometry (DI-ESI-MS; Waters Single Quadrupole Detector), and both 1-D proton and carbon NMR spectroscopy (Bruker/Ascend^TM^ 700 MHz with cryoprobe).

### Preparation and extraction of plant materials

Mature mangosteen (*G*. *mangostana* Linn.) fruits were collected from May to June at an orchard on private land (with consent of the owner; name included in the Acknowledgments section) in Felda Kampung Sertik (3.4826350, 102.0233610), Karak, Pahang, Malaysia. A herbarium voucher specimen UKMb40416 was prepared and deposited at the Herbarium of the Faculty of Science and Technology, Universiti Kebangsaan Malaysia. Dried *G*. *mangostana* pericarp (5 g) was weighed and then cut into small pieces. This material was soaked in 60 ml of either methanol or ethyl acetate (done in triplicate). The samples were then stirred at room temperature for 3 days and filtered using Whatman No. 1 filter paper. For the methanol extract, water (40 ml) was added to the filtrates followed by two steps of successive solvent partitioning with ethyl acetate. The combined ethyl acetate fractions were dried, weighed and assigned as the indirect ethyl acetate (IEA) extract (IEA). Mangosteen pericarp soaked directly and separately in ethyl acetate and methanol. The obtained extracts were dried, weighted and designated as the direct ethyl acetate (DEA) and methanol (DM) extracts. Finally, both the directly and indirectly obtained extracts (IEA, DEA, DM) were dissolved in acetonitrile (0.1 mg/ml) and filtered using 0.45 μm membrane filters for HPLC analysis.

### Instrumentation and HPLC conditions

The HPLC system (Agilent, series 1100, USA) used consisted of a quaternary pump, autosampler, solvent degasser, and diode array detector (DAD). The quantification wavelength of α-mangostin and samples were set at 254 nm and 330 nm. The chromatographic separation was performed at ambient temperature using an Atlantis T3 C18 column (2.1 mm x 150 mm, 100Å, 3 μm) was attached to a C18 guard column. The HPLC analysis was performed using a previously reported method [54] that was slightly modified to the following conditions: 70% acetonitrile and 0.1% (v/v) acetic acid diluted in ultra-pure water was delivered at the flow rate of 0.2 mL/min and run for 30 minutes. The sample injection volume was adjusted to 0.5 μl (0.5 μg). All solutions of mobile phase were freshly prepared, vacuum filtered through a 0.45 μm membrane and degassed by sonication for 20 min prior to use.

### Preparation of standard and calibration curve

A HPLC standard stock solution of α-mangostin (1 mg/mL) was prepared by dissolving the carefully weighed standard in an appropriate volume of acetonitrile and filtered using a 0.45 μm syringe filter and stored at 4°C until use. Serial dilution was performed thereafter to prepare (10, 50, 250,500 and 1000 μg/mL) for a calibration curve. Each standard solution was injected in triplicate (10 μL per injection) to enable further statistical analysis. The calibration curve was obtained by plotting the mean peak area versus the concentration of standard α-mangostin (μg/mL). Each calibration point was carried out in triplicate. Linearity (coefficient of determination, R^2^) of the calibration curve was determined by regression analysis. For intra-day intermediate precision, measurement repeatability was assessed by analysing the triplicate injections of the α-mangostin standard solution at five different concentrations (10, 50, 250, 500 and 1000 μg/mL) that were done within the same day. For inter-day intermediate precision, the same procedure was repeated on two different days. The precision was estimated from the relative standard deviation (RSD) and presented in term of % RSD of the peak area (n = 3) of the standard mixture. The limit of detection (LOD) and limit of quantification (LOQ) were calculated through the slope and standard deviation method [56] using the following formula:

LOD = (3.3 x δ)/S, and LOQ = (10 x δ)/S, where: δ is the standard deviation of the Y-intercept of the linear regression equations and S is the slope of the linear regression equations.

## Results and discussion

Standard α-mangostin was isolated from the directly obtained EA extract as a yellow compound that showed a [M-H]- base peak at m/z = 409 in the DI-ESI-MS (negative mode). The signals shown in the 1D ^1^H-NMR and ^13^C-NMR spectra (S2–S4 Figs) were consistent with values reported for α-mangostin in the literature [57].

**Fig 1:**
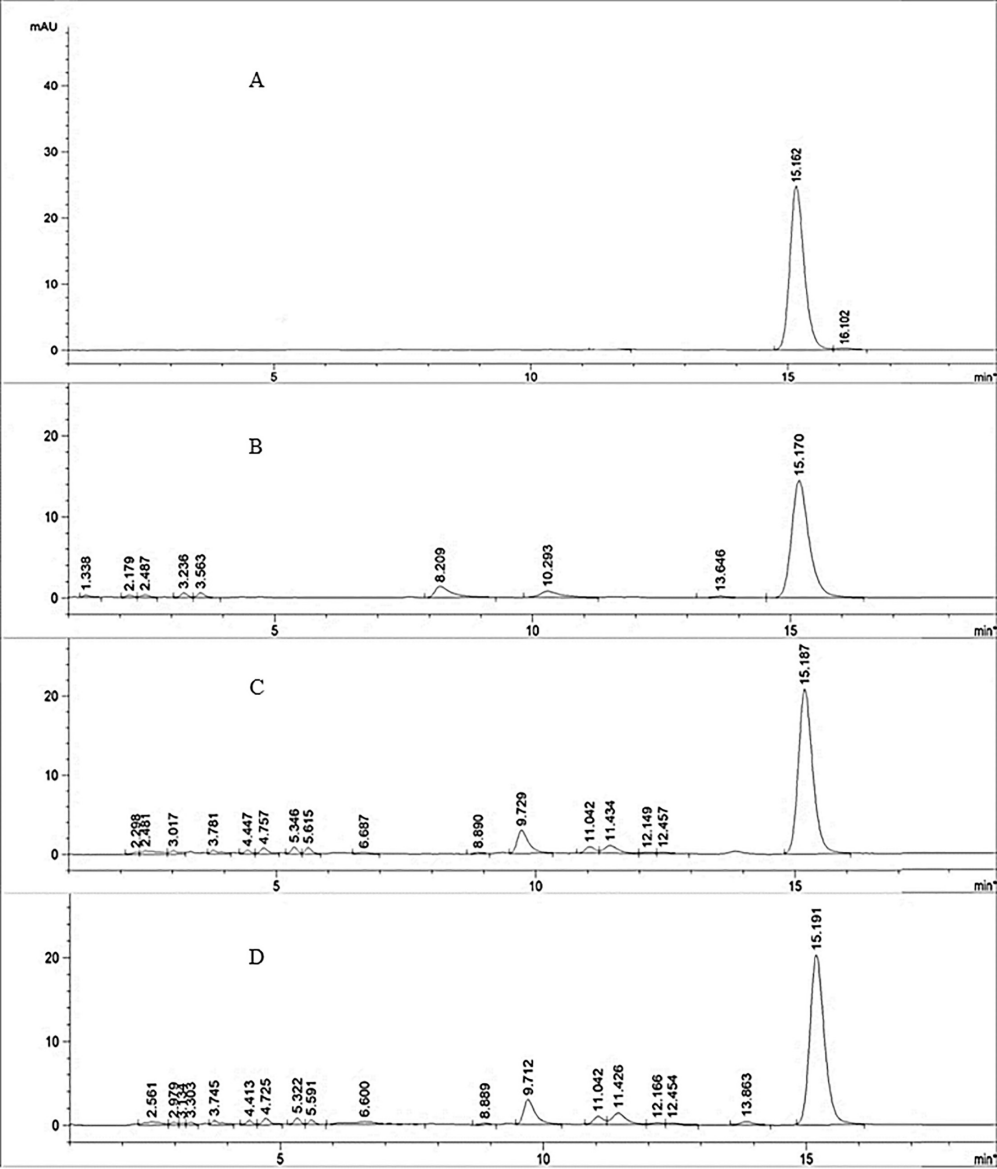
Chromatograms of the (A) α-mangostin standard, (B) MeOH direct extract (DM) (C) EA indirect extract (IEA)s (D) EA direct extract (DEA).

**Fig 2:**
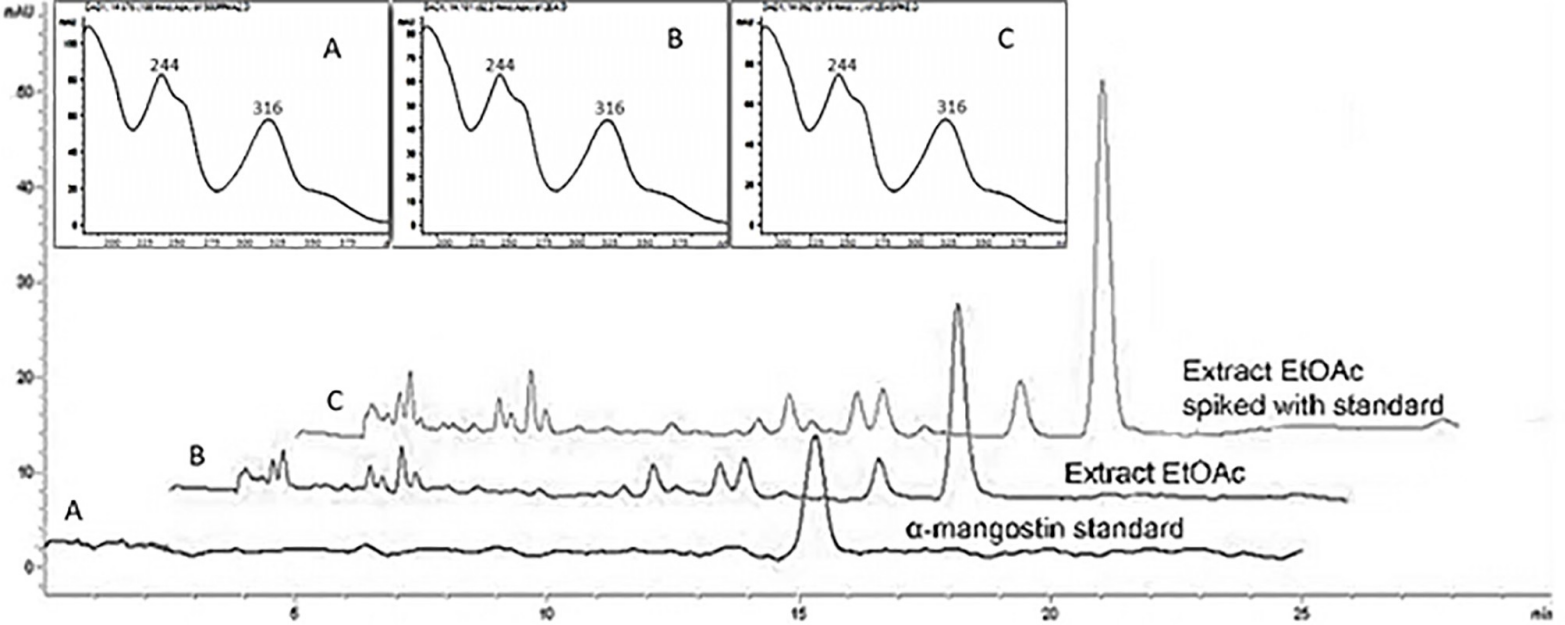
Chromatogram and UV Spectra of (A) α-mangostin standard, (B) α-mangostin peak in EA direct extract (DEA), and (C) DEA extract spiked with α-mangostin (0.5 mg/ml).

Based on HPLC profiles (Fig 1), the major peak in the chromatograms of all the extracts showed the same retention time as the reference α-mangostin standard that eluted at 15.1 minutes. The assignment of the major peak as α-mangostin was supported via spiking of the DEA extract with α-mangostin (0.5 mg/mL). The spiked chromatogram showed an increase in the intensity of the major peak. Additionally, the UV-Vis spectra of the major peak in the directly EA obtained extract before and after spiking showed the same profile as the α-mangostin standard (Fig 2).

The amount of α-mangostin from triplicate injections exhibited linearity over the evaluated range (S1 Data). The linear equations of the LOD and LOQ were y = 1205.5x -15.34 with LOD and LOQ of 61.9 ng and 187.7 ng, respectively. Linearity of the HPLC method was presented in terms of regression coefficient (R^2^) and was > 0.999 for the α-mangostin standard (S1 Fig). The % RSD of the peak area between intraday and interday precision was 0.36% which indicated good reproducibility. Likewise, the student’s t-test analysis showed no significant difference between intraday and interday precision, P > 0.01 (Table 1).

**Table 1:** Precision analysis of the HPLC method.

α-mangostin	Wavelength (nm)		*P* value (One-way ANOVA)
	254	330	
**^a^Intraday data**	0.87 ± 0.74	0.29 ± 0.27	0.45
**^a^Interday data**	0.51 ± 0.13	0.55 ± 0.49	1.00
**^b^*P* value (Student’s t-test)**	0.26	0.18	0.17

^a^The relative standard deviation of the peak area was calculated in the intraday and interday data, and the results are presented as average ± SD (n = 3).

^b^The *P* value was calculated by two tests: The One-way ANOVA to study the effect of the wavelength on the precision, and the Student’s t-test to study the difference between the intraday and interday data. Differences are considered significant at *P* < 0.01.

**Fig 3:**
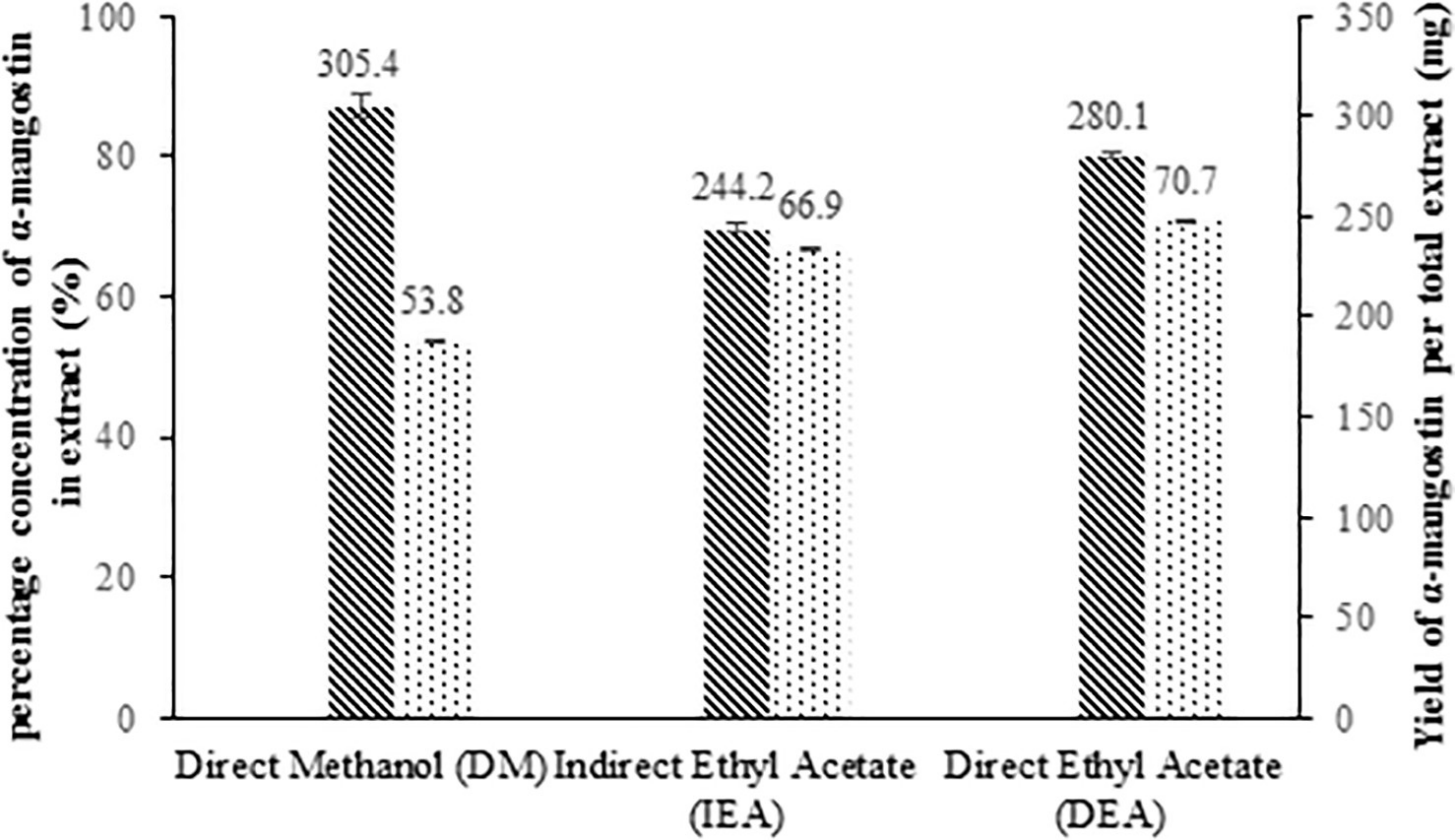
Comparison between concentration of α-mangostin in extract (%) and the yield of α-mangostin in total DM, IEA and DEA extracts (mg) in 5 g of dried pericarp.

Analysis of the number of peaks in the chromatograms of the different extracts (Fig 1) showed that more metabolites were present in both the DEA and IEA extracts (directly and indirectly) compared to the DM extract. However, the amount of DM extract obtained from the total dried pericarp used (5 g) was much higher (568.2 mg) than the DEA (396.0 mg) and IEA (365.0 mg) extracts (Table 2). This may be due to the higher abundance of non-xanthone compounds such as oligomeric polyphenolics in the DM extract that are non-/weakly UV active [58] compared to the DEA and IEA extracts. The percentage (% w/w) of α-mangostin content determined from the standard curve in the extracts (e) and dried pericarp (dp), were: DEA (70.7% e; 6.1% dp), IEA (66.9% e; 4.9% dp) and DM (53.8% e; 5.6% dp). Despite the differences in masses obtained for all the extracts, the amount of α-mangostin (mg) per total dried pericarp used (5 g) was similar in the DM (305.4 ± 5.8 mg) and DEA (280.1 ± 1.3 mg) extracts but was much lower in the IEA extract (244.2 ± 2.0 mg) (Fig 3) (S2 Data).

**Table 2:** Quantitative analysis of α-mangostin content using different extraction methods.

	Extracts		
	DM	IEA	DEA
**Total dried pericarp used (g)**	5.0	5.0	5.0
**Quantity of extract weighed (mg)**	568.2 ± 83.7	365.0 ± 88.2	396.0 ± 48.9
**Concentration of α-mangostin in extract (w/w)**	56.2 ± 8.9	49.1 ± 13.6	60.6 ± 7.8
**Yield of α-mangostin in total extract (mg)**	305.4 ± 5.8	244.2 ± 2.0	280.1 ± 1.3

DM = Direct Methanol extract, IEA = Indirect Ethyl Acetate extract, DEA = Direct Ethyl Acetate extract

In the literature, the extraction and isolation of α-mangostin from *G*. *mangostana* was mainly carried out using indirect extraction via solvent partitioning commonly involving chloroform or dichloromethane as exemplified by some recent reports [11, 52]. The use of direct extraction was less common particularly so with multiple solvents which is far more time-consuming. Although extraction based on halogenated solvents afforded good yields of α-mangostin [53, 59], our results indicate that comparable yields can be achieved with direct extraction using a single solvent with the more environmentally friendly and less toxic solvents methanol or ethyl acetate. Extraction using these green solvents would be more compliant with the needs of the pharmaceutical and food industries.

Although the DM extract showed the lowest concentration of α-mangostin (53.8% w/w) compared to the DEA (70.7% w/w) and IEA (66.9% w/w) extracts, it afforded the highest amount of extract. This corresponded to both the DM and DEA extracts showing the higher quantities of α-mangostin per total amount of dried pericarp used in this study (DM: 305.4 mg; DEA: 280.1 mg; IEA: 244.2 mg). Therefore, single-solvent direct extraction using the methanol or ethyl acetate is a better option than indirect extraction based on solvent partitioning for the extraction of α-mangostin. Furthermore, methanol or ethyl acetate are green solvents that can replace the halogenated solvents commonly used in many studies reporting the extraction and isolation of α-mangostin.

## Supporting information

S1 Data Calibration and precision data. (XLSX)

S2 Data Raw data-*Garcinia mangostana*. (XLSX)

S1 Fig Calibration curve of the α-mangostin standard. (DOCX)

S2 Fig DI-ESI-MS, negative mode of the α-mangostin standard. (DOCX)

S3 Fig 1H-NMR of isolated α-mangostin (acetone-d6, 700 MHz). (DOCX)

S4 Fig 13C-NMR of isolated α-mangostin (acetone-d6, 175 MHz). (DOCX)
