# Supplementary material for: From the Front or Back Door? Quantitative analysis of direct and indirect extractions of α-mangostin from mangosteen (Garcinia mangostana)
Source: PLoS One. 2018 Oct 15;13(10):e0205753. doi: 10.1371/journal.pone.0205753 (PMC6188793; doi:10.1371/journal.pone.0205753)
Supplement: S1 Fig — (DOCX) [file pone.0205753.s003.docx]

S1 Fig: Calibration curve of the α-mangostin standard.
