# Supplementary figures and images for: From the Front or Back Door? Quantitative analysis of direct and indirect extractions of α-mangostin from mangosteen (Garcinia mangostana)
Source: PLoS One. 2018 Oct 15;13(10):e0205753. doi: 10.1371/journal.pone.0205753 (PMC6188793; doi:10.1371/journal.pone.0205753)

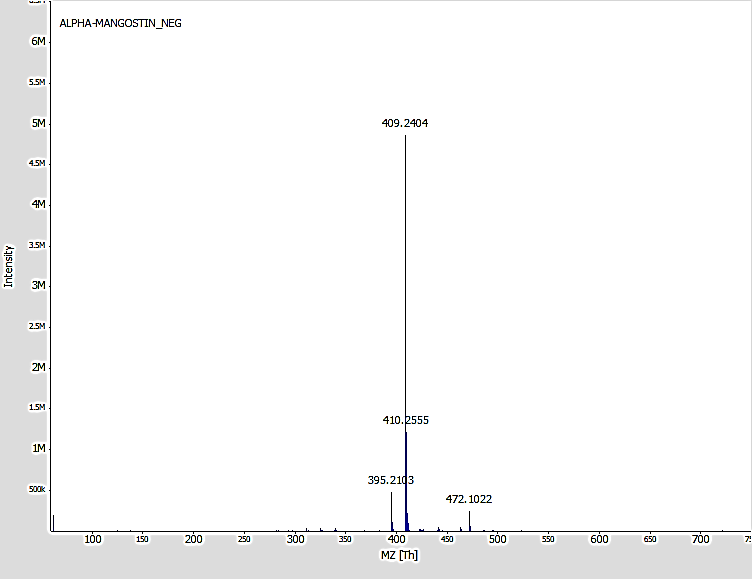


S2 Fig: DI-ESI-MS, negative mode of the α-mangostin standard

Supplement: S2 Fig — (DOCX) [file pone.0205753.s004.docx]

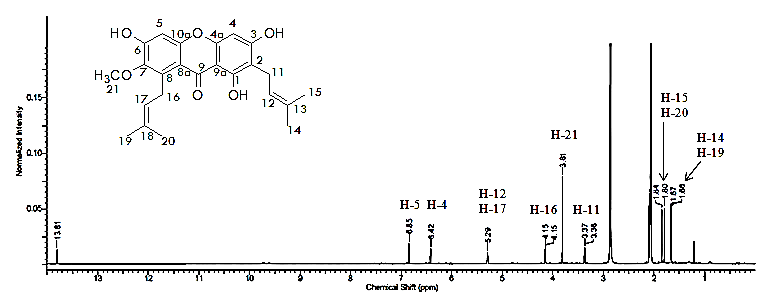


S3 Fig: 1H-NMR of isolated α-mangostin (acetone-*d6*, 700 MHz)

Supplement: S3 Fig — (DOCX) [file pone.0205753.s005.docx]

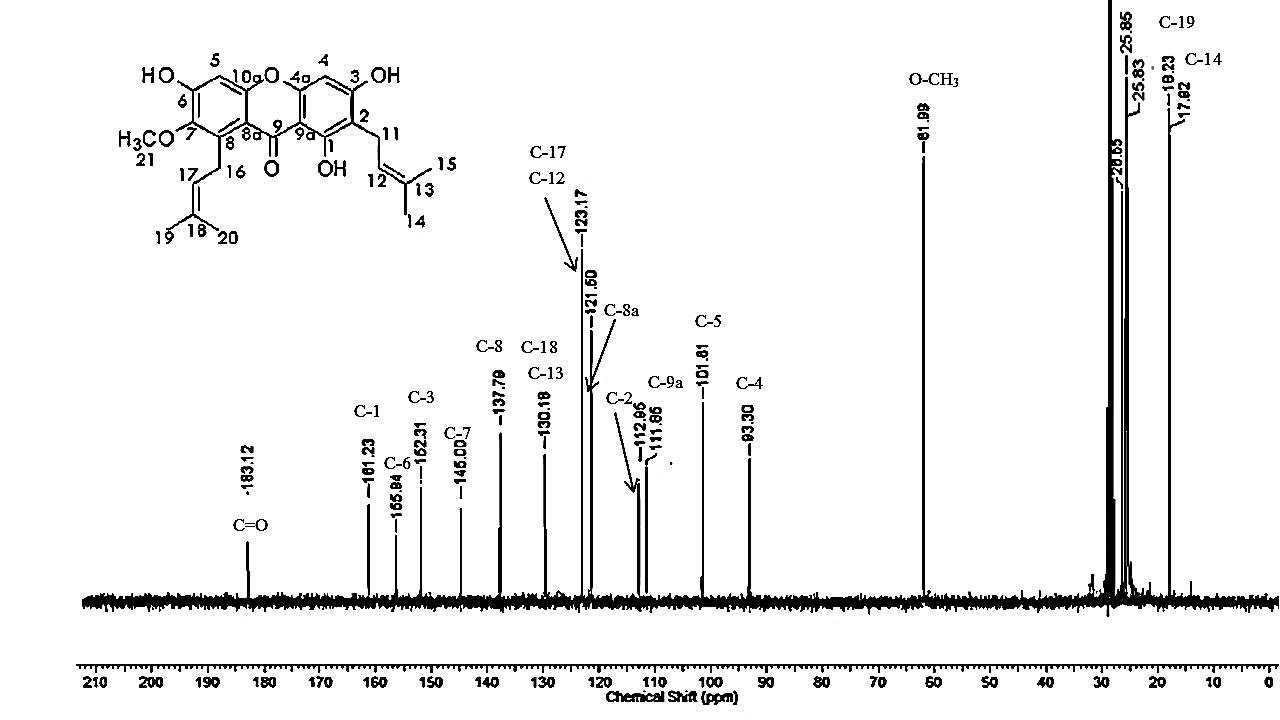


S4 Fig: 13C-NMR of isolated α-mangostin (acetone-*d6*, 175 MHz)

Supplement: S4 Fig — (DOCX) [file pone.0205753.s006.docx]
